# Fluoxetine Dose and Administration Method Differentially Affect Hippocampal Plasticity in Adult Female Rats

**DOI:** 10.1155/2014/123026

**Published:** 2014-03-17

**Authors:** Jodi L. Pawluski, Eva van Donkelaar, Zipporah Abrams, Virginie Houbart, Marianne Fillet, Harry W. M. Steinbusch, Thierry D. Charlier

**Affiliations:** ^1^GIGA-Neurosciences, University of Liège, 1 Avenue de l'Hôpital, Bâtiment B36, 4000 Liège, Belgium; ^2^School for Mental Health and Neuroscience, Department of Neuroscience, Faculty of Health, Medicine and Life Sciences, Maastricht University, Universiteitssingel 40, 6229 ER Maastricht, The Netherlands; ^3^Department of Biological Sciences, Irvine Hall, Ohio University, Athens, OH 45701, USA; ^4^Laboratory of Analytical Pharmaceutical Chemistry, Department of Pharmacy, CIRM, University of Liège, 1 Avenue de l'Hôpital, Bâtiment B36, 4000 Liège, Belgium

## Abstract

Selective serotonin reuptake inhibitor medications are one of the most common treatments for mood disorders. In humans, these medications are taken orally, usually once per day. Unfortunately, administration of antidepressant medications in rodent models is often through injection, oral gavage, or minipump implant, all relatively stressful procedures. The aim of the present study was to investigate how administration of the commonly used SSRI, fluoxetine, via a wafer cookie, compares to fluoxetine administration using an osmotic minipump, with regards to serum drug levels and hippocampal plasticity. For this experiment, adult female Sprague-Dawley rats were divided over the two administration methods: (1) cookie and (2) osmotic minipump and three fluoxetine treatment doses: 0, 5, or 10 mg/kg/day. Results show that a fluoxetine dose of 5 mg/kg/day, but not 10 mg/kg/day, results in comparable serum levels of fluoxetine and its active metabolite norfluoxetine between the two administration methods. Furthermore, minipump administration of fluoxetine resulted in higher levels of cell proliferation in the granule cell layer (GCL) at a 5 mg dose compared to a 10 mg dose. Synaptophysin expression in the GCL, but not CA3, was significantly lower after fluoxetine treatment, regardless of administration method. These data suggest that the administration method and dose of fluoxetine can differentially affect hippocampal plasticity in the adult female rat.

## 1. Introduction

Plasticity in the hippocampus has long been implicated in the etiology of mood disorders and regulation of stress [1–3]. Recent work has also suggested that the actions of antidepressant medications, such as selective serotonin reuptake inhibitors (SSRIs), on hippocampal plasticity may play an important role in alleviating symptoms of depression [4–6]. Serotonin, itself, is an important neurotransmitter involved in regulating the rate of hippocampal neurogenesis during adulthood, with lower levels of serotonin reducing the number of newly formed neurons in the hippocampus and elevated levels of serotonin increasing the rate of cell proliferation [7–11]. More importantly, increased serotonin levels, following SSRI treatment, significantly upregulate adult hippocampal neurogenesis [7, 12–14] and it has been suggested that the actions of SSRI medications on hippocampal plasticity (morphology and neurogenesis) may be important for alleviating the effects of stress on affect-related behaviours [4, 12, 15, 16].

Although there has been a substantial amount of work investigating the effects of SSRI medications on plasticity in the hippocampus and their role in treating depressive-like behavior, very little work has taken into account the effects of these medications on hippocampal plasticity in the adult female [17]. With women being 2-3 times more likely to suffer from depression, it is important to include females in research related to depression [18, 19]. Recent work focusing on females has shown that chronic administration of imipramine, a tricyclic antidepressant, to intact female rats exhibiting depressive-like behavior increases cell proliferation in the dentate gyrus [20]. However, others have shown that administration of fluoxetine, a popular SSRI, has no effect on cell proliferation or neurogenesis in the hippocampus of adult female rats [21, 22], but it does trigger spine formation in the hippocampus of the adult female rats [23].

More work is also needed to better mimic the clinical administration method of SSRIs. In humans, SSRIs are taken orally, usually one time per day. Unfortunately, the typical administration of antidepressant medications in rodent models of depression is invasive and stressful, with administration being done most often via injection, oral gavage, or minipump implant [24, 25]. Administration method alone affects the metabolism and effects of the medication [26, 27]. Interestingly, recent research in animal models investigating effects of environmental teratogens on development [28] has demonstrated that administration of a solution injected in a wafer cookie may be as effective as injecting a solution into the animal and thus would reduce the stress of the animal, particularly for long-term daily treatment (greater than 4 weeks). However, it remains to be determined whether this method of drug administration is effective for SSRIs and is comparable to other methods of antidepressant administration that are commonly used.

The aim of this project was to understand the role of fluoxetine treatment in hippocampal plasticity in the adult female rat, using two methods of SSRI administration. More specifically the proposed study aims to (1) determine the blood concentration levels of fluoxetine and its primary metabolite, norfluoxetine, in response to two administration types, cookie and minipump, at three fluoxetine doses (0, 5, and 10 mg/kg/day) and (2) determine the effect of these forms of fluoxetine administration on neural and synaptophysin expression in hippocampus. This study will provide important information toward the goal of generating a new and reliable method of antidepressant administration that eliminates the need for surgery, lowers the stress to the animal, can be readily applied to animal models, provides accurate drug levels, and most closely models antidepressant administration in humans. This work will result in a more accurate understanding of the neurobiological and physiological impact of SSRIs in preclinical models, ultimately improving our understanding of how to treat mood disorders in humans.

## 2. Material and Methods

### 2.1. Animals

Thirty-six intact adult female Sprague-Dawley rats (250–300 g; Charles River Laboratories, France) were used in the present study. Rats were kept under standard laboratory conditions in a 12 h:12 h light/dark schedule (lights on at 07:00 h), initially housed in pairs in clear polyurethane bins (48 cm × 27 cm × 20 cm) with* ad libitum* access to rat chow (Sniff) and tap water. Females were randomly divided into two conditions (cookie or minipump) with three dose options: (1) fluoxetine (10 mg/kg/day), (2) fluoxetine (5 mg/kg/day), and (3) vehicle (0 mg/kg/day), for a total of 6 groups. Females were weighed weekly and individually housed with standard enrichment once treatment began. All experiments were approved by the Animal Ethics Board of Maastricht University in accordance with Dutch governmental regulations (DEC 2010-146). All efforts were made to minimize the pain and stress levels experienced by the animals.

### 2.2. Fluoxetine Administration

#### 2.2.1. Cookie Treatment

On days 1-2, females in the cookie treatment groups were trained for oral ingestion of the medication treatment. For training, females were fed 1/9th of a vanilla wafer cookie (Crousti fondante, Delacre, Belgium), filled with saline [29]. After cookie training, females in the cookie groups were fed a cookie filled with fluoxetine (Fagron, Belgium: 5 mg/kg or 10 mg/kg) dissolved in vehicle (25% propylenediol in saline) or vehicle solution once per day between 8:00 and 9:00 am After cookie feeding females were monitored to ensure that cookies were eaten. Cookies were consistently eaten by all females. Females were treated with fluoxetine or vehicle for 14 days.

#### 2.2.2. Minipump Treatment

Females in the minipump treatment group were administered fluoxetine or vehicle via osmotic minipumps (Alzet Osmotic pumps, 2ML2, Charles River, The Netherlands) for 2 weeks. Minipump implants were filled with either fluoxetine (Fagron, Belgium: 5 mg/kg/day or 10 mg/kg/day) dissolved in vehicle (25% propylenediol in saline) or with vehicle as previously described [22, 30–33]. Minipumps were implanted subcutaneously in the dorsal region, while females were under mild isoflurane anesthesia. The weight of a full 2ML2 minipump was approximately 7.5 g. Thirty minutes prior to the minipump implant, the NSAID carprofen (dose: 2.5–5 mg/kg) was given subcutaneously for pain. Implantation took a maximum of 20 minutes.

### 2.3. Glucose Levels

At sacrifice, glucose levels were taken from trunk blood (mg/dL). The first drop of blood was removed; the second drop was placed on a test strip (GLUCOCARD X-SENSOR test strips) for immediate glucose measurement with a hand-held glucose meter (GLUCOCARD TM X-meter, A. Menarini Diagnostics, Benelux, N.V., Valkenswaard, The Netherlands).

### 2.4. Blood Collection

To determine serum levels of fluoxetine and its active metabolite, norfluoxetine, blood collection, via the tail vein, from females treated with fluoxetine was taken twice on days 6 and 10 between 8-9 am, after cookie feeding, and 2–4 pm. Blood from vehicle-treated females was taken on day 6 between 8-9 am only. At decapitation, trunk blood was also taken from all animals between 1–3 pm. Blood samples were stored at 4°C overnight and centrifuged at 10,000 g for 10 minutes. Serum was collected and stored at −80°C until analysis.

### 2.5. Estradiol Levels

To investigate whether estradiol levels affected measures of cell proliferation, 17*β*-estradiol (E2) was measured in a subset of animals that were randomly selected (18 in total). All samples were run in duplicate using commercially available 17*β*-oestradiol (E2) I^125^ radioimmunoassay (RIA) kits from MP Biomedicals (MP Biomedicals, Belgium). The average intracoefficient of variation for the assay is 2.75% for the E2 assay. The lowest detection limit for E2 was 1.4 pg/mL.

### 2.6. Fluoxetine and Norfluoxetine Determination

Drug concentrations were determined from serum using liquid chromatography coupled with mass spectrometry (LC-Chip-MS/MS) that was used as previously described [22, 33, 34]. Briefly, the chromatographic separation was achieved on a 1200 series LC-chip system (Agilent Technologies, Germany) using an ultrahigh capacity chip including a 500 nL trapping column and a 150 mm × 75 *μ*m analytical column, both packed with a Zorbax 80SB 5 *μ*m C18 phase (Agilent Technologies). The mobile phase was composed of H_2_O/FA (100 : 0.1, v/v) (A) and ACN/H_2_O/FA (90 : 10 : 0.1, v/v/v) (B) and used in gradient elution mode. Mass spectrometric detection was performed using a 6340 ion trap equipped with a nanoelectrospray ionization source operating in positive mode (Agilent Technologies, Waldbronn). Finally, an Oasis *μ*Elution MCX 96-well plate (Waters, UK) was used to prepare the samples for the analysis. All conditions were performed in duplicate and back-calculated using a calibration curve. Fluoxetine and norfluoxetine levels were averaged across days to provide one morning and one afternoon value.

### 2.7. Histology

All histological procedures were based on previous work [35, 36]. Fourteen days after treatment, females were deeply anesthetized with sodium pentobarbital, weighed, and rapidly decapitated. Following extraction, the right hemisphere of the brains was stored at 4°C in 4% paraformaldehyde for 24 h, then cryoprotected in 30% sucrose/phosphate-buffered saline solution for up to one week, frozen on dry ice, and kept at −80°C. The left hemisphere was stored and not used in the present investigation. Brain tissue was sliced in 40 *μ*m sections on a cryostat (Leica). Tissue was stored in brain antifreeze solution and maintained at −15°C until use. The level of cell proliferation in the granule cell layer and subgranular zone (GCL/SGZ) of the hippocampus was assessed using an endogenous marker for cell proliferation, Ki67. Every 6th section throughout the right hippocampi was stained as previously described [35, 37]. Sections were blocked with H_2_O_2_ and incubated overnight in rabbit anti-Ki67 (1:500; Vector Laboratories) or blocked with H_2_O_2_ and NGS and incubated overnight in mouse antisynaptophysin (1:500; Sigma Aldrich). Sections were then incubated for 2 h in biotinylated donkey anti-rabbit (1:500; Jackson Immunoresearch Laboratories, West Grove, PA) or biotinylated goat anti-mouse (1:200 Vector BA-9200) secondary antibody. Brain sections were further processed by using the avidin-biotin complex (ABC Elite kit; 1:1000; Vector laboratories, USA). DAB (3,3-diaminobenzidine) Peroxidase Substrate Kit (SK-4100, Vector Laboratories) was used as a substrate to obtain a color reaction. Sections were mounted on gelatin-coated slides and dried overnight, dehydrated, stained with cresyl violet (Ki67-ir only), and coverslipped with PermountTM (Fisher Scientific, USA).

#### 2.7.1. Ki67 Quantification

**Figure 1:**
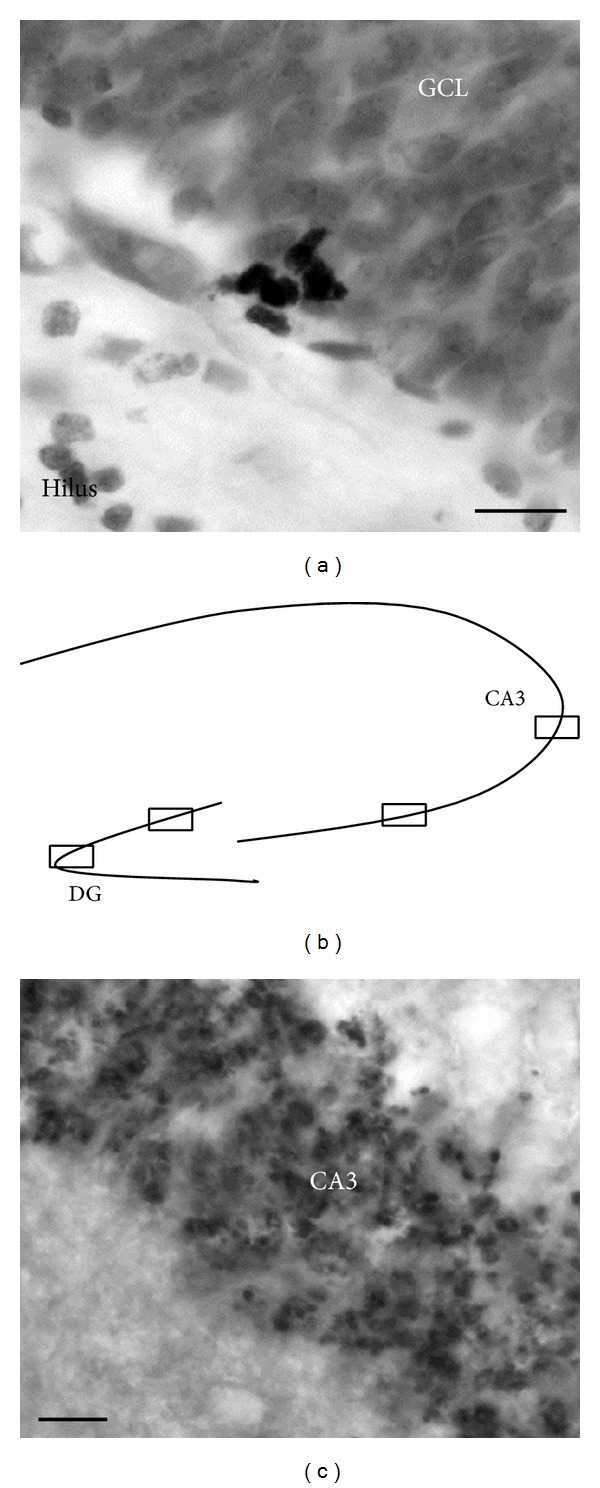
(a) Photomicrograph representing Ki67 immunoreactive cells in the dentate gyrus. (b) Drawing of hippocampal areas selected for quantification of synaptophysin expression and (c) a photomicrograph representing synaptophysin immunohistochemistry in the hippocampus (40x). Scale bar = 10 *μ*m.

The number of Ki67 immunoreactive (-ir) cells in granule cell layer/subgranular zone (GCL/SGZ) was counted under 40x objective using a Nikon Microphot SA microscope. Cells were considered Ki67-ir if they were intensely stained and exhibited medium round or oval nuclear bodies. Ki67-ir cells were counted on half of every 6th section throughout the entire hippocampus. For representative Ki67-ir cells in the GCL/SGZ of the hippocampus, see Figure 1.

#### 2.7.2. Synaptophysin Quantification

Three dorsal sections of the hippocampus, located between stereotaxic coordinates bregma −2.64 mm to −4.92 mm [38], were analysed per animal for synaptophysin-immunoreactivity by an observer blind to conditions. Photomicrographs were taken for two areas within the CA3 and GCL/SGZ of the hippocampus from each of the three sections (e.g., see Figure 1) for a total of 6 photomicrographs per area. Immunoreactivity was examined under 40x objective using a Nikon Microphot SA and Nikon DS-Qi1MC camera with Nikon NIS Elements F4.00 software. The software ImageJ64 (Wayne Rasband, NIH, Bethesda, MD, USA) was used for quantification of optical densities of synaptophysin. The relative optical density was defined as the difference between optical density (grey level) measures after calibration within the area of interest and in an equivalent adjacent area (background). For representative photomicrographs of synaptophysin density, see Figure 1.

### 2.8. Statistical Analysis

The fluoxetine and norfluoxetine levels were analyzed using repeated-measures analysis of variance tests (ANOVA) with administration type (cookie versus minipump) and dose (0, 5, or 10 mg/kg) as the between-subjects factors. Repeated measure ANOVAs were also used to assess synaptophysin density in the CA3 and GCL/SGZ. ANOVAs were conducted on the total number of Ki67 cells in the GCL, the percent change in Ki67-ir cells from controls, body weight, and glucose levels with administration type (cookie versus minipump) and dose (0, 5, or 10 mg/kg) as the between-subjects factors. Any effects of estradiol on the number of Ki67-ir and synaptophysin-ir cells were controlled for. Post hoc comparisons utilized the Fisher's LSD procedure. Statistical significance was set at *P* ≤ 0.05.

## 3. Results

### 3.1. Fluoxetine and Norfluoxetine Levels

**Figure 2:**
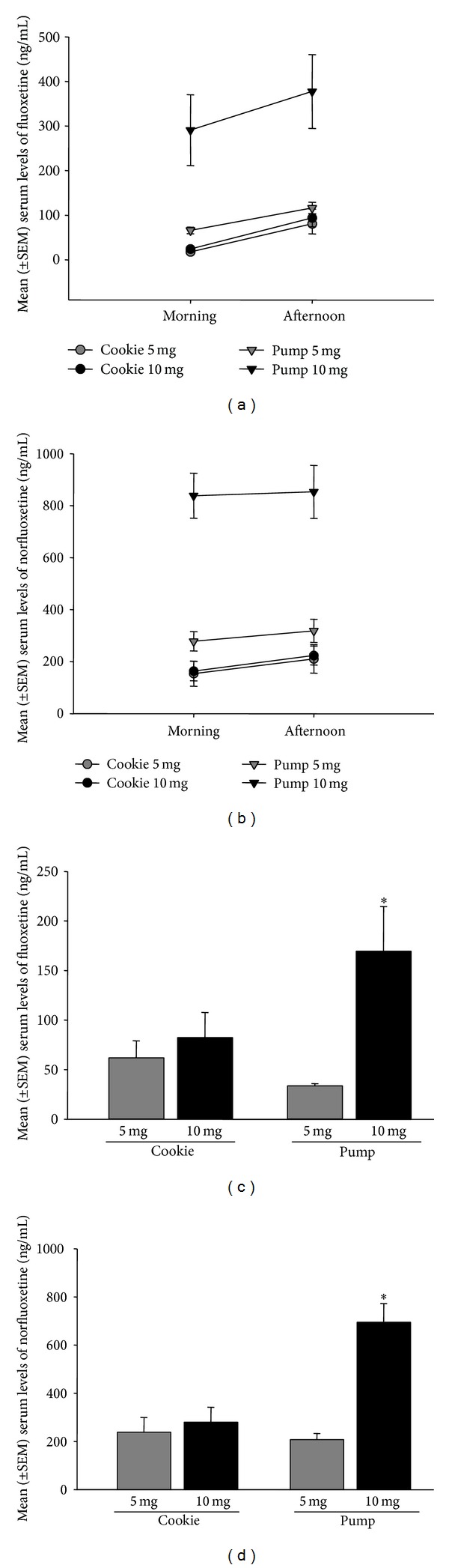
Mean (±SEM) serum levels of fluoxetine (a, c) and norfluoxetine (b, d) (ng/mL). (a, b) There were significantly higher serum levels of fluoxetine/norfluoxetine in the animals receiving 10 mg/kg/day of fluoxetine via minipump. Serum levels were significantly lower in the morning and were significantly higher in the minipump group (0.000001 < *P* < 0.015). (c, d) At sacrifice, serum from trunk blood revealed significantly higher levels of fluoxetine/norfluoxetine in the animals receiving 10 mg/kg/day of fluoxetine via minipump (0.00001 < *P* < 0.03). (*n* = 6/group). ∗∗ denotes 10 mg significantly different from all other groups.

For serum fluoxetine and norfluoxetine levels there was a significant interaction between administration type (cookie, minipump) and dose (5 mg/kg/day, 10 mg/kg/day) (fluox: *F*(1,20) = 7.95, *P* = 0.012, norfluox: *F*(1,20) = 21.16, *P* = 0.0002) with significantly higher serum levels of fluoxetine/norfluoxetine in the animals receiving 10 mg/kg/day of fluoxetine via minipump (0.000001 < *P* < 0.0005; Figure 2). There was also a main effect of time (fluox: *F*(1,20) = 57.70, *P* = 0.000001, norfluox: *F*(1,20) = 7.14, *P* = 0.015) with morning levels of fluoxetine/norfluoxetine being significantly lower than afternoon levels. There was a main effect of administration method with minipump administration of fluoxetine resulting in significantly higher serum levels of fluoxetine/norfluoxetine compared to cookie administration (fluox: *F*(1,20) = 14.68, *P* = 0.001, norfluox: *F*(1,20) = 43.46, *P* = 0.000001). There was also a significant main effect of dose on norfluoxetine levels (*F*(1,20) = 22.98, *P* = 0.00011).

At sacrifice, serum from trunk blood revealed significantly higher levels of fluoxetine/norfluoxetine in the animals receiving 10 mg/kg/day of fluoxetine via minipump (0.00001 < *P* < 0.03; interaction effect fluox: *F*(1,20) = 4.52, *P* = 0.046, norfluox: *F*(1,20) = 14.06, *P* = 0.0013, Figure 2). There were also main effects of dose (fluox: *F*(1,20) = 8.29, *P* = 0.0093, norfluox: *F*(1,20) = 19.84, *P* = 0.00024) and a main effect of administration type (norfluox: *F*(1,20) = 10.44, *P* = 0.0042) on drug levels in trunk blood at sacrifice. There were no other main or interaction effects (0.24 < *P* < 0.91).

As expected, vehicle-treated animals did not have detectable serum levels of fluoxetine or norfluoxetine.

### 3.2. Ki67-ir Cells

**Figure 3:**
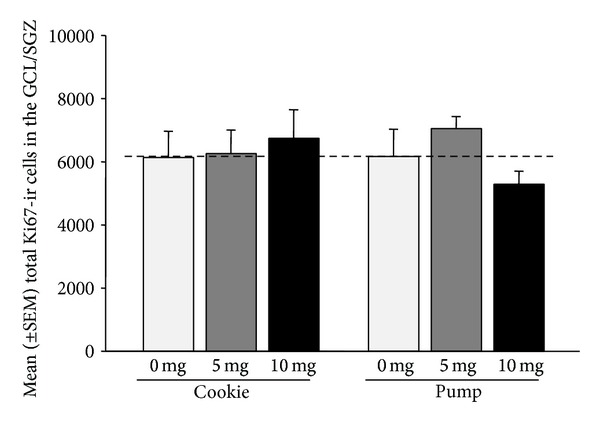
Mean (±SEM) total number of Ki67-ir cells in the GCL/SGZ. Minipump animals receiving the 5 mg/kg/day dose of fluoxetine had significantly more Ki67-ir cells in the GCL compared to minipump animals receiving the 10 mg/kg/day dose, when looking at overall change from baseline (control) (*P* = 0.013). (*n* = 5-6/group). Dashed line indicates baseline.

Minipump animals receiving the 5 mg/kg/day dose of fluoxetine had significantly more Ki67-ir cells in the GCL compared to minipump animals receiving the 10 mg/kg/day dose, when looking at overall change from baseline (controls) (*F*(1,9) = 9.64,  *P* = 0.013; *n* = 5/group). There were no other significant effects of fluoxetine dose or administration type on total number of Ki67-ir cells in the GCL/SGZ (0.3 < *P* < 0.7; Figure 3). There was also no effect of estradiol levels on number of Ki67-ir cells in the GCL/SGZ (*P* = 0.98).

### 3.3. Synaptophysin Density

**Figure 4:**
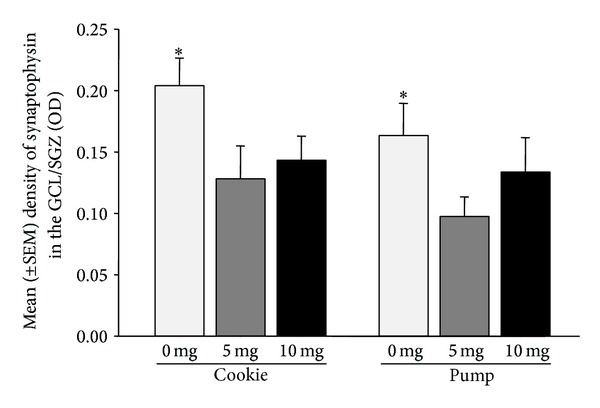
Mean (±SEM) synaptophysin expression in the GCL/SGZ of the hippocampus. Synaptophysin density was significantly greater in vehicle-treated animals, regardless of administration method (0.000001 < *P* < 0.03). (*n* = 6/group). ∗ denotes 0 mg significantly different from 5 mg and 10 mg.

Synaptophysin density in the GCL/SGZ was significantly greater in vehicle-treated animals, regardless of administration method (0.000001 < *P* < 0.03, Figure 4; region (CA3, GCL) by dose interaction for synaptophysin density: *F*(2,30) = 8.48, *P* = 0.0012). Synaptophysin density was also significantly greater in the GCL/SGZ than in the CA3 (main effect of region: *F*(1,30) = 40.35, *P* = 0.000001). There were no other significant main or interaction effects between groups in synaptophysin density and no effect of estradiol levels on synaptophysin density (0.15 < *P* < 0.99; Table 1).

### 3.4. Glucose Levels

As expected, animals fed with the cookie had significantly elevated blood glucose levels compared to animals with minipump implants (*F*(1,30) = 8.0435, *P* = 0.008; Table 2); however, these levels were still within the physiological range. There were no other significant differences between groups (*P* < 0.82).

### 3.5. Body Weight

For body weight measurements there was no significant main effect of administration type or dose on overall change in weight (0.29 < *P* < 0.58).

## 4. Discussion

Findings of the present study show that two weeks of fluoxetine treatment results in a significant decrease in synaptophysin expression in the dentate gyrus, but not the CA3 region, of adult female rats. In turn, we found that administration method (cookie versus minipump) and fluoxetine dose differentially affected hippocampal cell proliferation, with only females receiving the 5 mg/kg dose via a minipump having increased cell proliferation in the GCL compared to females receiving the 10 mg/kg dose via a minipump. Furthermore, administration method differentially affected circulating levels of fluoxetine and its active metabolite, norfluoxetine, with the greatest levels of fluoxetine being evident at a 10 mg/kg dose administered via minipump.

### 4.1. Fluoxetine Effects Synaptophysin Expression in the Dentate Gyrus

In the present study, fluoxetine treatment significantly decreased synaptophysin expressionin the dentate gyrus and had no effect on synaptophysin expressionin the CA3 region of the hippocampus. To our knowledge there is no previous research on the effects of fluoxetine treatment on synaptophysin expression in adult female rats. However, previous research in adult male rats has shown that 7 days of fluoxetine treatment significantly increases synaptophysin mRNA levels in the granule cell layer of the hippocampus [39]; however, they did not measure protein levels. Work in hippocampal cell culture, under toxic conditions, and in male Ts65Dn mice (model of Down Syndrome) also shows that fluoxetine can rescue or improve synaptophysin expression [40, 41]. In addition, the decrease in synaptophysin expression in the dentate gyrus of female rats with fluoxetine administration is perhaps counterintuitive given the general idea that SSRI medication enhances hippocampal neurogenesis [6, 42]. However, it is well documented in males only that SSRI medications increase hippocampal plasticity and alleviate depressive-like behaviors [43], suggesting a significant role of estradiol and progesterone on the effects of SSRI medications in adult females. Further neurochemical and behavioural data are needed to fully understand the functional significance of SSRIs on hippocampal plasticity in the adult female.

In the present study we did not find a significant effect of fluoxetine treatment of synaptophysin expression in the CA3 region of the hippocampus. Previous work has shown that fluoxetine administration (5 mg/kg) significantly increases pyramidal spine formation in both the CA1 and CA3 regions of the hippocampus of adult female rats [23], with effects in the CA1 region being evident after 5 days of fluoxetine administration and effects in the CA3 region being evident after 2 weeks of fluoxetine administration [23]. However, previous work in adult male rats shows no effect of fluoxetine treatment (10 mg/kg) on synaptophysin mRNA density [39]. Discrepancies between the present study and the previous work in females may be due to methodological techniques as Hajszan et al. [23] administered fluoxetine via intraperitoneal injections and used electron microscopy to quantify spine densities, whereas, in the present study, fluoxetine was administered via a cookie or minipump and synaptophysin immunohistochemistry was measured. Furthermore, Hajszan et al. [23] used ovariectomized female rats whereas female rats in the present study were cycling. Estradiol alone is known to have marked effects on spine density, particularly in the CA1 region of the hippocampus [44–47], and has been shown to increase serotonin metabolite levels in the CA3 region of the hippocampus [48]. In addition, estradiol has been shown to upregulate serotonin synthesis in the dorsal raphe in a similar manner to fluoxetine [49]. Recent work suggests that the effects of fluoxetine on spine density in adult female rats are only evident in ovariectomized females and when the endogenous actions of estradiol on hippocampal spine density are disturbed [50]. Thus, there are likely marked interactions between circulating estradiol levels and the effects of fluoxetine on spine density in the hippocampal formation.

### 4.2. Fluoxetine Dose and Administration Effect on Cell Proliferation

In the present study, we found effects of fluoxetine on hippocampal cell proliferation only after administration of fluoxetine via minipump. Here we show that after 2 weeks of minipump administration females receiving the 5 mg/kg dose of fluoxetine had significantly more proliferating cells in the GCL compared to females receiving the 10 mg/kg via minipump. This work shows that fluoxetine levels can differentially affect cell proliferation in the adult female rat. These findings also replicate previous work showing that fluoxetine (5 mg/kg) has no effect on cell proliferation in the hippocampus of adult female rats when compared to controls [21, 22]. Interestingly, previous work in adult male rats shows that 2 weeks of fluoxetine administration (7 mg/kg) via minipump, as in the present study, increases hippocampal cell proliferation [26]. This work and a previous one [21] point to marked sex differences in the effect of fluoxetine on hippocampal plasticity. Sex/gender must be taken into consideration when investigating neurobiological effects of SSRI medications, particularly as these medications are more often used to treat depression in women [19]. Apart from sex/gender, exposure to stress and changes in circulating corticosterone levels also play an important role in the effects of fluoxetine on hippocampal neurogenesis [22, 26]. For example, previous work shows the effect of fluoxetine treatment on hippocampal neurogenesis in the adult female is markedly increased in females exposed to stress [22].

### 4.3. Effects of Administration Method on Fluoxetine

In the present study fluoxetine administration method differentially affected measures of hippocampal plasticity and also serum fluoxetine and norfluoxetine levels. Here we show that serum fluoxetine and norfluoxetine levels, in a dose of 5 mg/kg, do not markedly differ between cookie and minipump administration. However, serum fluoxetine and norfluoxetine levels, after a fluoxetine dose of 10 mg/kg, were significantly elevated in animals treated with a minipump. This difference between administration methods in serum levels of fluoxetine at a higher dose is likely due to metabolism of fluoxetine by cytochrome P450 enzymes in the liver after oral (cookie) administration [51], thus leading to lower circulating levels of fluoxetine and norfluoxetine. In higher doses it may be advantageous to administer fluoxetine twice a day in a cookie in order to increase the circulating levels of fluoxetine and norfluoxetine. We have recently used a twice-a-day cookie administration method (total fluoxetine dose 10 mg/kg) during the postpartum period and shown significant effects on offspring development [33]. Others have also shown that voluntary fluoxetine administration in a cookie dough ball is an effective and noninvasive technique to chronically administer this, and potentially other, medication [52]. Thus, voluntary SSRI medication administration is possible and may be a valuable way to further understand how these medications affect neurobiological processes.

## 5. Conclusions

The present study investigated the effect of fluoxetine, a common SSRI antidepressant medication, on hippocampal plasticity in the adult female rat. Main findings show that two weeks of fluoxetine treatment results in a significant decrease in synaptophysin expression in the dentate gyrus, but not the CA3 region, of the hippocampus. In turn, administration method (cookie versus minipump) and fluoxetine dose (5 mg versus 10 mg) differentially affected hippocampal cell proliferation in the GCL, with the females receiving the 5 mg/kg dose of fluoxetine via minipump having significantly more proliferating cells compared to females receiving the 10 mg/kg via minipump (when investigating overall change from baseline). Furthermore, administration method differentially affected circulating levels of fluoxetine and its active metabolite, norfluoxetine, with the greatest levels of fluoxetine being evident with a 10 mg/kg dose given via minipump.

## Figures and Tables

**Table 1:** Mean (±SEM) synaptophysin expression in the CA3 region of the hippocampus (optical density) (*n* = 6/group).

Fluoxetine dose/day	Cookie treatment	Pump treatment
0 mg/kg	0.1129 ± 0.009	0.0798 ± 0.017
5 mg/kg	0.1100 ± 0.019	0.0875 ± 0.014
10 mg/kg	0.0994 ± 0.019	0.0996 ± 0.024

**Table 2:** Mean (±SEM) blood glucose levels at sacrifice (mg/dL). As expected, animals fed with cookies had significantly elevated blood glucose levels compared to animals with minipump implants (*P* = 0.008,  *n* = 6/group).

Fluoxetine dose/day	Cookie treatment	Pump treatment
0 mg/kg	12.53 ± 1.80	9.27 ± 0.52
5 mg/kg	11.28 ± 1.46	9.25 ± 0.59
10 mg/kg	12.92 ± 1.94	9.22 ± 0.60
